# ID2 promotes tumor progression and metastasis in thyroid cancer

**DOI:** 10.1007/s12020-023-03674-3

**Published:** 2024-01-10

**Authors:** Zhongming Deng, Min Xu, Zhenghua Ding, Jianqiao Kong, Juanjuan Liu, Zelin Zhang, Ping Cao

**Affiliations:** 1 Department of General Surgery, Xiangyang No.1 People’s Hospital, Hubei University of Medicine, Xiangyang, 441000 China; 2 Department of Anesthesiology, Xiangyang No.1 People’s Hospital, Hubei University of Medicine, Xiangyang, 441000 China; 3 Department of Oncology Department, Xiangyang No.1 People’s Hospital, Hubei University of Medicine, Xiangyang, 441000 China

**Keywords:** ID2, PI3K/Akt pathway, EMT, Stemness, Thyroid cancer

## Abstract

**Background:**

Inhibitor of DNA Binding 2 (ID2) plays a crucial role in tumor cell proliferation, invasion, metastasis, and stemness. Aberrant ID2 expression is associated with poor prognosis in various cancers. However, the specific function of ID2 in thyroid cancer remain unclear.

**Method:**

The TCGA database were utilized to explore the clinical relevance of ID2 in cancer. GO, KEGG, and TIMER were employed to predict the potential roles of ID2 in cancer. Functional analysis, including CCK-8, colony formation, transwell, wound healing, and sphere formation experiments, were conducted to determine the biological functions of ID2 in human cancers. Western blot (WB), RT-qPCR, and immunohistochemical (IHC) analyses were used to investigate the relationship between ID2 and downstream targets.

**Results:**

Our study revealed significant overexpression of ID2 in various malignant tumor cells. Knocking ID2 significantly inhibited cancer cell proliferation and invasion, while overexpressing ID2 enhanced these capabilities. Additionally, ID2 mediates resistance of cancer cells to protein kinase B (or Akt) inhibitions. Further WB and IHC experiments indicated that ID2 promotes the phosphorylation activation of phosphatidylinositol 3-kinase (PI3K)/Akt signaling pathway, thereby upregulating the expression of downstream proliferation, epithelial-mesenchymal transition (EMT), and stemness-related markers.

**Conclusion:**

We found that ID2 significantly promotes thyroid cancer cell proliferation, migration, EMT, and stemness through the PI3K/Akt pathway. Moreover, ID2 plays a crucial role in regulating cancer immune responses. It may serve as a potential biomarker for enhancing the efficacy of chemotherapy, targeted therapy, and immunotherapy against cancer.

## Background

Thyroid cancer is the most common malignancy disease of the endocrine system and ranks fifth in the world in terms of new cases in 2020 [1, 2]. While the incidence of the majority of cancers is static or decreasing, the incidence of thyroid cancer is increasing at an accelerated rate. Despite the seemingly favorable prognosis of thyroid cancer, 30–40% of patients experience early lymph node metastasis, leading to a 50% reduction in the 10-year survival rate. Therefore, it is crucial to study the molecular mechanisms and key regulatory factors underlying the progression and prognosis of thyroid cancer, in order to identify new biomarkers for personalized prevention and treatment.

Inhibitor of DNA Binding 2 (ID2) belongs to the ID family of members and is a helix-loop-helix transcription regulator. Its role involves the regulation of crucial biological processes such as cell differentiation, proliferation, and apoptosis. ID proteins are often overexpressed in cancer stem cells, where they regulate the formation of embryonic and adult tissue stem cells. For instance, in prostate cancer, androgens may exert control over proliferation, apoptosis, and tumor suppression through ID1, ID3, ID2, and ID4 [3, 4]. ID1 overexpression contributes to invasion and tumor progression [5]. Furthermore, overexpression of ID1 and ID3 leads to reduced expression of cell cycle inhibitors p16, p21, and p27, resulting in increased cell proliferation [6]. There are also reports linking ID proteins to chemoresistance in cancer cells. ID1 and ID3 regulate the self-renewal of colon cancer stem cells through p21. Concurrent knockout of ID1 and ID3 enhances the sensitivity of cancer stem cells to the chemotherapy drug oxaliplatin [7]. Related research suggests that ID2 may be dysregulated and play varying roles in the progression of several cancer types. However, the function of ID proteins in thyroid carcinoma remains unknown.

Epithelial-Mesenchymal Transition (EMT) [8] plays a pivotal role in the initial steps of cancer progression involving migration and invasion. It results in the loss of epithelial cell polarity, detachment of cells from the basement membrane, and enables cancer cells to undergo tumorigenesis and metastasis [9, 10]. During EMT, E-cadherin [11] is downregulated, facilitating cancer cell dissociation, while N-cadherin [12] is upregulated, promoting angiogenesis. Moreover, various transcription factors are involved in EMT, such as Snail [13] and Sox2 [14], which regulates multiple transcriptional targets to promote the acquisition of migratory and invasive properties. Additionally, they contribute to cancer cells acquiring stem-like characteristics (stemness). Researches indicate that the induction of EMT facilitated by ID2 promotes invasion and metastasis in hepatocellular carcinoma (HCC) [15]. The upregulation of ID2 enhanced N-cadherin expression while suppressing E-cadherin leves, thereby participating in the EMT process and hepatic metastasis in colorectal cancer (CRC) [16]. MMP9 (Matrix Metalloproteinase 9) [17, 18] is an enzyme that plays a critical role in extracellular matrix degradation and remodeling processes. Excessive activation of MMP9 can lead to abnormal degradation of the extracellular matrix, contributing to the development of inflammation, tumor invasion, and metastasis. Research has indicated that constitutive expression of ID2 in prostate cancer cells can promote an invasive phenotype [3]. However, the relationship between ID2, EMT, and tumor stemness of thyroid cancer remains unclear.

PI3K/Akt pathway [19, 20] is intricately associated with the initiation and progression of tumors, as well as the development of treatment resistance and recurrence. The activation of PI3K leads to the phosphorylation and activation of Akt. Activated Akt promotes tumor growth through various pathways, including the resistance to apoptosis, the promotion of cell cycle, the facilitation of EMT, and the modulation of metabolism, thereby supporting the malignant behavior of tumor cells. Studies have reported that patients with papillary thyroid carcinoma (PTC) harboring the BRAF ^V600E^ mutation exhibit increased aggressiveness, and aberrant activation of the PI3K/Akt pathway is associated with an elevated risk of disease-specific mortality [21]. Additionally, mutations in PIK3CA, AKT1, and PTEN have been observed in aggressive and recurrent thyroid tumors [22–24], indicating that the PI3K/Akt pathway plays a pivotal role in the initiation of thyroid tumors, second only to the MAPK signaling pathway. Recent research has unveiled that ID2 regulated the growth and stemness of pancreatic cancer through the PI3K/Akt pathway, modulating key stemness factors, Nanog and Sox2 [25]. ID2 promotes the progression of CRC [25] by activating the PI3K/Akt signaling pathway, thereby enhance migration and invasion.

In this study, we observed an upregulation of ID2 expression in thyroid carcinoma (THCA) and its correlation with patient survival. Further analysis revealed that ID2 promotes THCA proliferation, migration, EMT, and stemness through the PI3K/Akt signaling pathway. Collectively, our findings suggest that ID2 may serve as a potential biomarker and therapeutic target for THCA.

## Materials and methods

### Cell culture and transfection

Nthy-ori 3-1 was human thyroid epithelial cell, TPC1 and CAL-62 were human thyroid cancer cell lines. They were obtained from American Type Culture Collection (ATCC). TPC1 and CAL-62 were cultured in DMEM (Gibco, Darmstadt, Germany) supplemented with 10% Fetal Bovine Serum (FBS, Gibco, Darmstadt, Germany), Nthy-ori 3-1 was cultured in 1640 (Gibco, Darmstadt, Germany) supplemented with 10% FBS. They were cultured in a humidified atmosphere of CO_2_ /air (5%/95%) at 37 °C. TPC1 and CAL-62 cells were transduced with lentivirus (knockdown ID2, over-expression ID2 and control) using co-transfection reagent P (Genechem, Shanghai, China). ID2 shRNA sequence [26]: GCCTACTGAATGCTGTGTATACTCGAGTATACACAGCATTCAGTAGGC.

### Real-time fluorescence quantitative PCR (RT‐qPCR)

Total RNA was extracted from the samples with TRIzol (Vazyme, Nanjin, China). The complementary DNA was synthesized using a PrimeScript RT Reagent Kit (Takara Bio, Otsu, Japan), mRNA expression was examined by real-time quantitative polymerase chain reaction (RT-PCR) using SYBR Green Master Mix (Vazyme, Nanjin, China) and performed in ABI StepOne Plus Real-time PCR Detection System (Applied Biosystems, Foster City, CA). The expression level of GAPDH was simultaneously quantified as a standard control. The sequences of all primers were purchased from Sangon (Shanghai, China) and listed below.

ID2 (F 5′- CCGTGAGGTCCGTTAGG -3′; R 5′- TGAGCTTGGAGTAGCAGTCG -3′), CDK4 (F 5′- TTGCGGCCTGTGTCTATGGT -3′; R 5′- ATCAAGGGAGACCCTCACGC -3′), CDK6 ′(F 5′- TGCGGAGAGCCGACTGAC -3′; R 5′- TTCCCTCCTCGAAGCGAAGT -3′), Cyclin D1 (F 5’- GCTGCGAAGTGGAAACCATC -3′; R 5′- CCTCCTTCTGCACACATTTG -3′), SOX2 (F 5′- GGACAGTTACGCGCACATGA -3′; R 5′- AGCCGTTCATGTAGGTCTGC -3′), MMP9 (F 5′- TTTGAGTCCGGTGGACGATG -3′; R 5′- GCTCCTCAAAGACCGAGTCC -3′), CDH1 (F 5′- GTCCTGGGCAGACTGAATTT -3′; R 5′- GACCAAGAAATGGATCTGTGG -3′), CDH2 (F 5′- TGGACCATCACTCGGCTTA -3′; R 5′- ACACTGGCAAACCTTCACG -3′), GAPDH (F 5′- TGACATCAAGAAGGTGGTG -3′; R 5′- TCCACCACCCTGTTGCTGT -3′).

### Primary antibodies and drug treatment

The rabbit normal IgG and antibodies against ID2 (1:1000, A0996), Snail (1:1000, A5243), Phospho-AKT1-S473 (AP0140), c-MYC (1:1000, A19032) were purchased from ABclonal (Wuhan, China). The rabbit normal IgG and antibodies against E-cadherin (1:1000, 20874-1-AP), N-cadherin (1:1000, 22018-1-AP), MMP9 (1:1000, 10375-2-AP), PI3 Kinase p110 alpha (1:1000, 67071-1-Ig), GAPDH (1:2000, 10494-1-AP), Ki67 (1:2000, 27309-1-AP), Pan-keratin (1:2000, 26411-1-AP), and HRP-conjugated secondary antibody (1:10000, SA00001-2) was purchased from proteintech (Shanghai, China). The rabbit normal IgG and antibodies against CDK4 (1:1000, D9G3E), CDK6 (1:1000, D4S8S), cyclin D1 (1:1000, E3P5S), AKT (1:1000, 4691 S) were purchased from CST (Shanghai, China). phospho-PI3 kinase p85/p55 (Tyr467/Tyr199) (1:1000, HY-P80846) and Sox2 (1:1000, HY-P80334) was purchased from MedChemExpress (MCE, Shanghai, China).

Thyroid cancer cells treated with Akt inhibitor MK-2206 (HY-108232) or Akt agonist Recilisib (HY-101625), which were purchased from MCE (Shanghai, China). After 48 h drug treatment, cell proteins were extracted. After concentration normalization, western blot experiments were conducted.

### Cell viability and proliferation

The viability of cells in 96-well plates (1000 cells/well) was tested using Cell Counting Kit 8 (CCK-8, Beyotime, Shanghai, China). CCK-8 reagent was diluted by serum free media (1:100, 100 µL) and added to each well of 96-well plate. The plates were incubated in 37 °C degree for 1 h. The absorbance was measured at 450 nm using a microplate reader (BioTek, VT). The proliferation tests were carried out for five days continuously.

For traditional, 1 × 10^3^ cells/well were seeded per well in 6-well plate. After 10–14 days, seeding plates were fixed with 4% paraformaldehyde for 30 min and then stained with crystal violet for 30 min. The colony number and size were calculated by Image J (version: 2.1.0/1.53c).

### Cell migration

2 × 10^4^ cells were seeded into the upper chambers of transwell culture plates (Corning, Shanghai, China). 500 μl culture medium supplemented with 20% FBS was put into the lower chambers. After incubation for 24 h for migration assays, cells penetrated to the lower surface of the membrane were fixed with 4% paraformaldehyde for 30 min and then stained with crystal violet for 30 min and counted.

The cells were seeded into 6-well-plates. When the cells cover the entire well, wounded the cells with 200 μL sterile pipette tips. After washing off the floating cells with phosphate buffer solution (PBS), the cells were cultured in medium with 1% FBS. The photos were taken under the microscope at 0, 8, and 24 h after wound and calculated by formula: Wound Healing size % = (width_0 h_ - width_x h_) / width_0 h_ * 100%, (x = 8 or 24).

### Animal experiments

BALB/c-nu mice (4–5 weeks famale mice) were purchased from Gempharmatech Co., Ltd. (Jiangsu, China), fed in a special pathogen-free (SPF) animal facility and allowed to eat and drink ad libitum. The mice were randomly assigned to 3 groups with 5 mice per group and then subcutaneously inoculated with sh-ID2, oe-ID2 or NC cell suspensions. Approximately 1 × 10^7^ cells in 100 µL of serum-free 1640 medium were injected directly into subcutaneous fat. When palpable tumors formed (or after 28 days), animals were anesthetized using tribromoethanol (Aibei Biotechnology, Nanjing, China), and euthanized by dislocated their cervical vertebrae. Later, xenograft tumors were harvested, and tumor weight and size were measured.

Tissues were washed with PBS and with 3% H_2_O_2_ for 5–20 min. Then, the tissues were washed with PBS after dipping in distilled water. Citrate buffer was applied to perform antigen retrieval at 100 °C for 25 min. Tissues were then incubated at 4 °C overnight respectively with rabbit antibody against ID2, Ki-67, pan-keratins, CDK4, CDK6, Cyclin D1, Snail, E-cadherin and N-cadherin. The next day, after washing with PBS, the tissues were treated in Biotin-conjugated Affinipure Goat Anti-Rabbit IgG(H + L) (1:200, SA00004-2, proteintech) at room temperature for 1 h, washed with PBS and immersed in 500 μl of a diaminobenzidine working solution at room temperature for 20 min. Finally, the slides were counterstained with hematoxylin and mounted in crystal mount medium.

### Bioinformatics analysis and statistical analysis

RNA sequencing data were downloaded from the Cancer Genome Atlas Database (TCGA) (https://portal.gdc. cancer.gov/). Genome-wide ID1, ID2, ID3, ID4 expression profiles patients were downloaded from TCGA and University of California Santa Cruz (UCSC) Xena [27] (https://xenabrowser.net/). Tumor IMmune Estimation Resource (TIMER) [28, 29] database (https://cistrome.shinyapps.io/timer/) was utilized to assess the expression levels of ID proteins in tumor tissue and adjacent normal tissue of thyroid cancer patients. Kaplan-Meier (KM) curve and receiver operating characteristic (ROC) curve analysis was performed by SPSS 22.0 (SPSS Inc., Chicago, IL, USA), and ploted by GraphPad prism 9 software (GraphPad software, Inc., La Jolla, CA). Thyroid cancer samples from TCGA-THCA were divided into high-expression and low-expression groups based on the median level of ID2. The R package t.test was employed to assess the differential significance for each gene between the high- and low-expression groups. P. adjust function was utilized to compute the false discovery rate (FDR) for each gene, thereby obtaining comprehensive differential information for the gene. Upregulated and downregulated differential genes were subjected to Kyoto Encyclopedia of Genes and Genomes (KEGG) and Gene ontology (GO) enrichment analysis. The KEGG Restore API I (https://www.kegg.jp/kegg/rest/keggapi.html) was employed to acquire the most recent gene annotations for KEGG Pathways. Gene annotations for GO were obtained from the “org.Hs.eg.db” R package (version 3.1.0). Enrichment analysis was performed using the “clusterProfiler” R package (version 3.14.3) to attain results on gene set enrichment. The criteria set for analysis were a minimum gene set of 5, a maximum gene set of 5000, and statistical significance defined as a *P* value < 0.05 and a false discovery rate (FDR) < 0.25. ID2 binding molecules and network were analyzed and ploted by search tool for the retrieval of interacting genes/proteins (STRING, Version 12.0, https://cn.string-db.org/) [30]. Expression correlation between ID2-binding molecules and ID2 were obtained from TCGA database and ploted by Gene Expression Profiling Interactive Analysis (GEPIA, http://gepia.cancer-pku.cn/) [31].

Parametric data are shown as means ± standard deviations (SDs) and nonparametric data as medians and ranges. Two-way ANOVA or one-way ANOVA with Tukey’s multiple comparison test was used for multiple group analysis. Unpaired Student’s t-tests were used to compare data between two groups. Two-tailed *P* values < 0.05 were considered statistically significant. Statistical analyses were performed using GraphPad prism 9 software (GraphPad software, Inc., La Jolla, CA) and SPSS 22.0 (SPSS Inc., Chicago, IL, USA).

## Results

### Differential expression and clinical features analysis

**Fig. 1:**
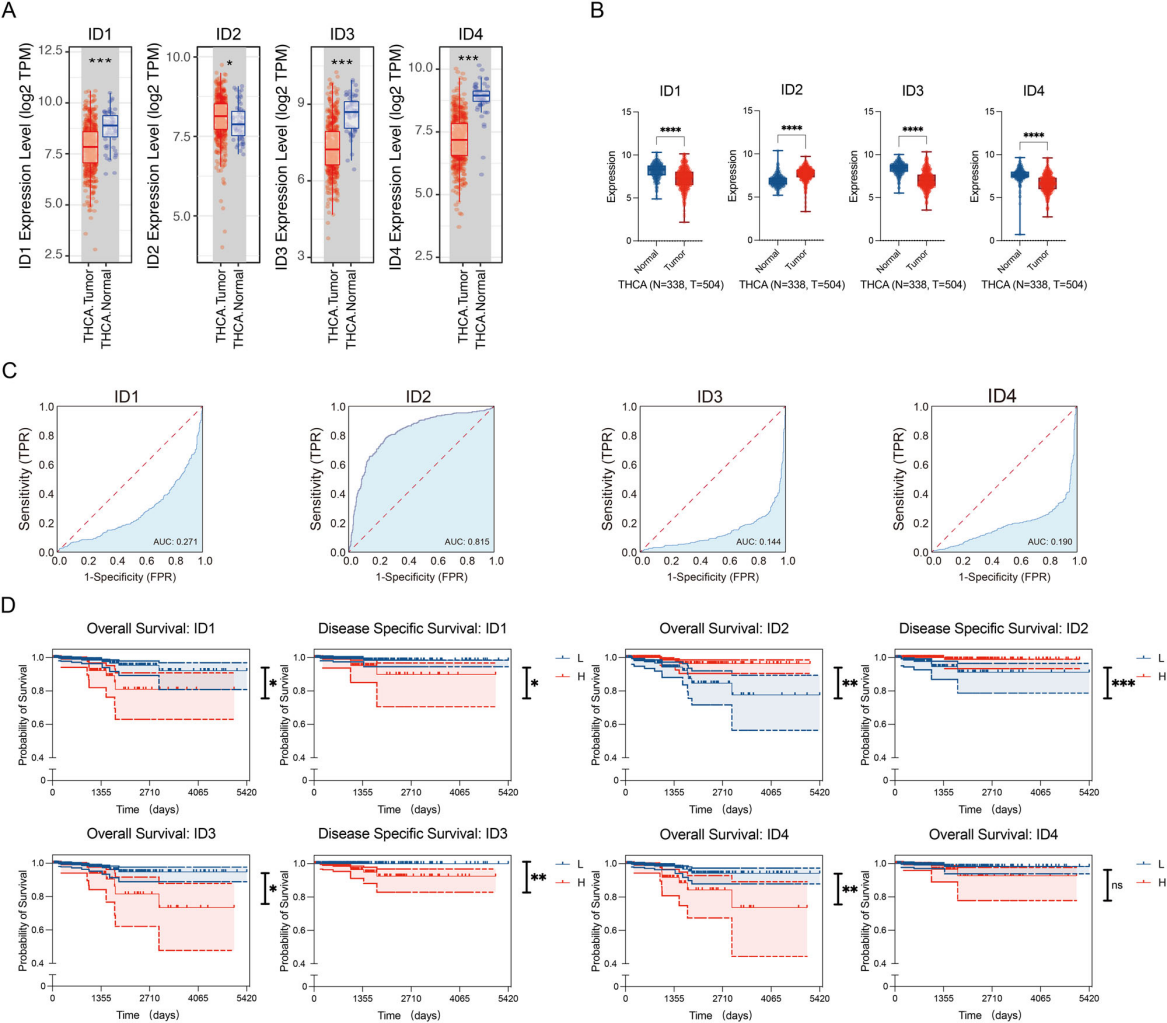
Association of ID2 expression with clinical characteristics. **A** Differential expression of ID2 in thyroid cancer tissues and adjacent normal tissues based on TIMER. **B** Expression profile of ID2 in tumor specimens compared to paired normal tissues in TCGA and GTEx datasets. **C** ROC curve depicting the efficiency of ID2 expression levels in distinguishing thyroid cancer from non-tumor controls. **D** Correlation between OS, DSS, and the expression of ID proteins genes in thyroid cancer. *p < 0.05, **p < 0.01, ***p < 0.005, ****p < 0.001

The TIMER database was used to evaluate the expression of the ID family (ID1, ID2, ID3, ID4) in tumor tissues and adjacent normal tissues of thyroid cancer (Fig. 1A). The expression of ID2 was significantly higher in tumor compared to normal tissues. Analyses using the TCGA and GTEx databases from USCS Xena also showed higher ID2 expression in thyroid cancer versus normal tissues (Fig. 1B). We obtained 28 PTC patients and 16 healthy controls from the GEO database (GSE138198), and found much higher levels of ID2 expression in thyroid tumors than normal controls (*p* = 0.0022, Fold change = 3.034). However, the expression levels of ID1, ID3, and ID4 were downregulated in thyroid cancer (Fig. 1B). Taken together, ID2 was upregulated and may serve as a biomarker or target in thyroid cancer.

The diagnostic utility of the ID family in thyroid cancer was examined using ROC curve analysis. ID2 (AUCs = 0.815) had a high degree of accuracy in the diagnosis of thyroid cancer, while ID1, ID3, and ID4 had poor diagnostic performance (Fig. 1C). We analyzed the association between ID2 expression and clinical pathological outcomes. We found no significant differences in ID2 expression levels between female/male, young (≤60 years)/elderly (>60 years), early (I + II)/advanced (III + IV) stage, no (N0)/presence (N1) of lymph node metastasis, and no (M0)/presence (M1) of distant metastasis in thyroid cancer patients (Table 1). Besides, in thyroid cancer, higher expression of ID2 was related to shorter overall survival (OS) and disease-specific survival (DSS) (Fig. 1D). Based on the above results, we hypothesized that thyroid cancer incidence and prognosis are strongly associated with ID2.

**Table 1:** The relationship between the expression of ID2 and various clinicopathological variables in the TCGA database

Characteristics	Total	ID2 expression		*P* value
		Low	High	
Total	568	284	284	
Age (years)				0.116
≤60	432	208	224	
>60	136	76	60	
Gender				0.851
Male	156	77	79	
Female	412	207	205	
T-stage				0.589
T1	154	77	77	
T2	189	91	98	
T3	196	104	92	
T4	27	11	16	
N-stage				0.742
N0	258	132	126	
N1	255	127	128	
M-stage				0.71
M0	327	161	166	
M1	11	5	6	
Stage				0.947
I	321	160	161	
II	59	31	28	
III	125	61	64	
IV	61	32	29	
Size				0.076
≤1 cm	61	34	27	
>1 cm, ≤3 cm	299	136	163	
>3 cm	114	94	208	

### Functional enrichment and immune infiltration analysis of ID2

**Fig. 2:**
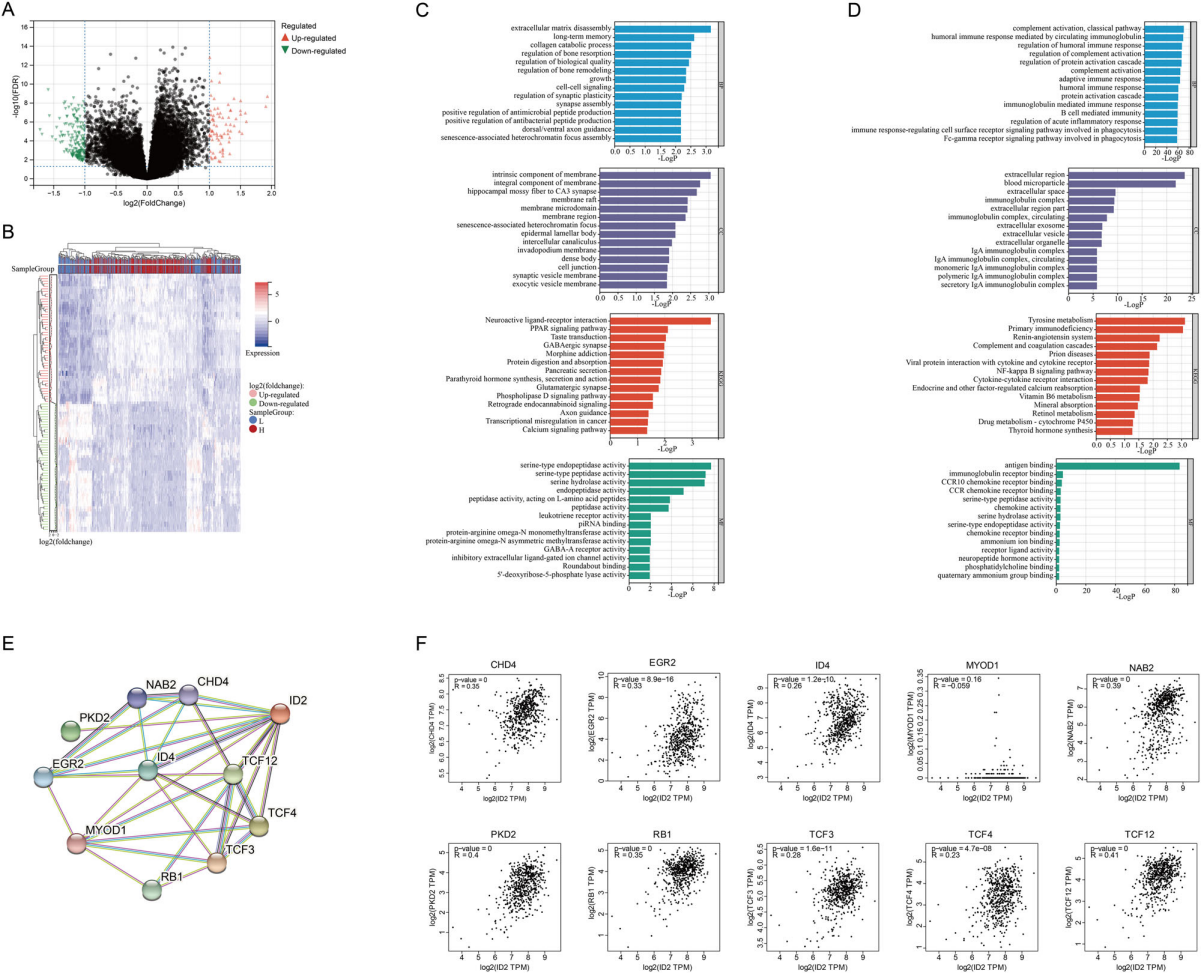
The potential function of ID2 in thyroid cancer. Volcano plot (**A**) and heatmap (**B**) depicting the significant different expressed gene analysis between high and low ID2 level groups. GO and KEGG analyses examining upregulated (**C**) or downregulated (**D**) significant differentially expressed genes. **E** Protein-protein interaction (PPI) analysis of ID2-relating molecules. **F** Correlation analysis exploring the relationship between the gene expression of ID2 and ID2-relating molecules

Thyroid cancer cases in TCGA were divided into high and low ID2 expression groups based on the median value, and a total of 77 upregulated genes and 187 downregulated genes were identified (*p* < 0.05, twofold difference) (Fig. 2A, B). GO and KEGG analyses were performed to assess the functions of these genes (Fig. 2C, D). Differential genes upregulated were enriched in serine-type endopeptidase activity, and neuroactive ligand-receptor interaction pathway. While downregulated genes were enriched in antigen binding, the tyrosine metabolism pathway. According to the protein-protein interaction (PPI) network, CHD4, EGR2, ID4, MYOD1, NAB2, PKD2, RB1, TCF3, TCF4, and TCF12 were identified as ID2-relating molecules. In addition, we examined the mRNA expression relationship between ID2 and ID2-relating molecules in thyroid cancer. Except for MYOD1, ID2 had a positive correlation with the relating molecule in mRNA expression level (Fig. 2E, F).

### ID2 promoted proliferation, migration and EMT in thyroid cancer

**Fig. 3:**
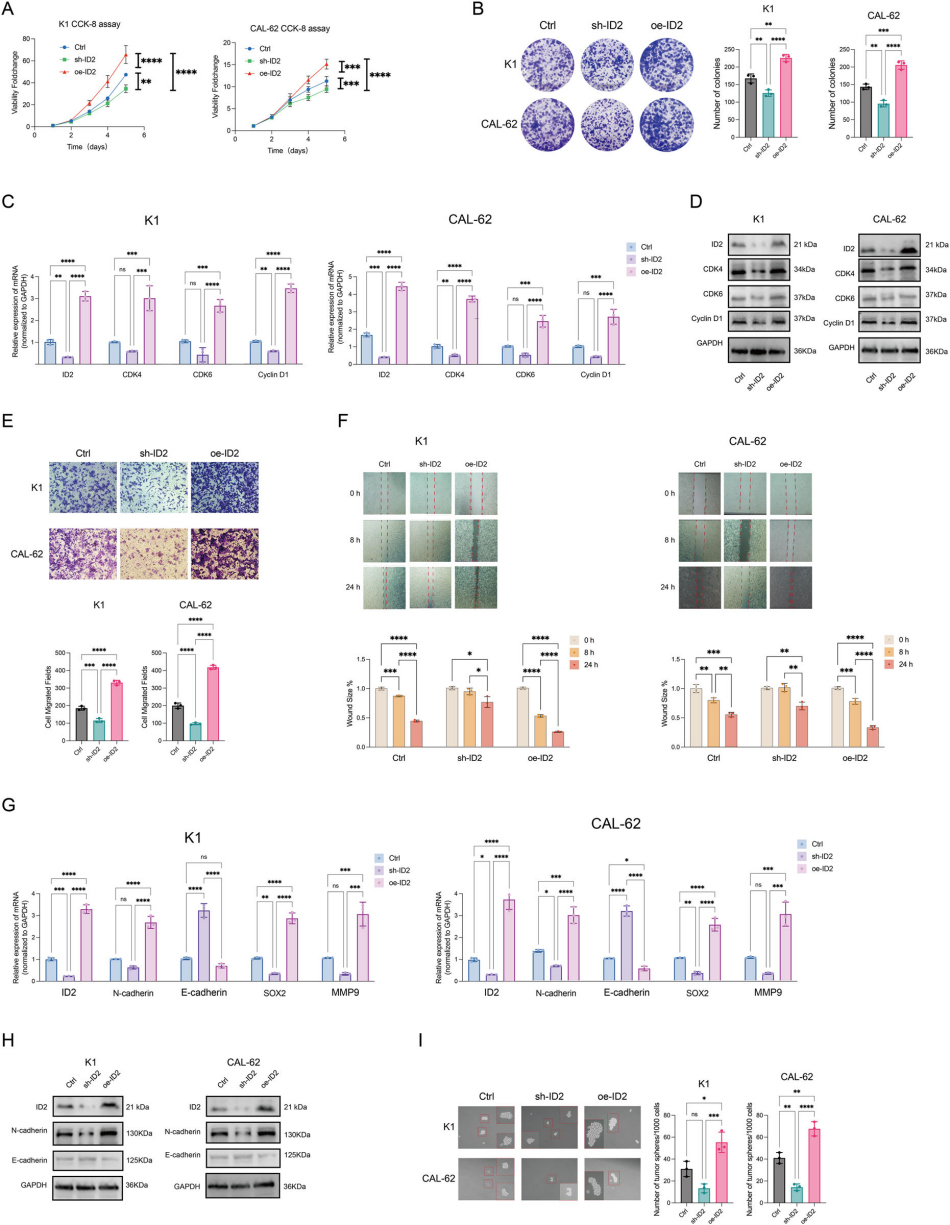
ID2 promotes proliferation, migration, and spheroid formation of thyroid cancer cells in vitro. Cell proliferation capacity of K1 and CAL-62 cells transfected with sh-ID2, control, or oe-ID2 assessed by CCK-8 assay (**A**) and colony formation assay (**B**) after transfection. Changes in mRNA (**C**) and protein (**D**) expression levels of proliferation-related markers in K1 and CAL-62 cells transfected with sh-ID2, Ctrl, or oe-ID2. Transwell (**E**) and scratch healing (**F**) assays conducted on K1 and CAL-62 cells transfected with si-ID2, Ctrl, or oe-ID2 to assess migration ability. Changes in mRNA (**G**) and protein (**H**) expression levels of EMT-related markers in K1 and CAL-62 cells transfected with sh-ID2, Ctrl, or oe-ID2. **I** Spheroid formation experiment of K1 and CAL-62 cells transfected with si-ID2, Ctrl, or oe-ID2. * *p* < 0.05, **p < 0.01, ***p < 0.005, ****p < 0.001

ID2 was highly expressed in two thyroid cancer cell lines (CAL-62 and K1) compared to normal human thyroid epithelial cells (Nthy-ori 3-1) (Fig. S1A). After knockdown and overexpression ID2 in K1 and CAL-62, RT-qPCR and WB were used to confirm the efficacy of ID2 expression regulation (Fig. S1B, C). Over-expression ID2 promoted the proliferation, while ID2 knockdown inhibited the viability and clonogenicity compared to control (Fig. 3A, B). We speculated that ID2 may regulate the cell cycle molecules in thyroid cancer. WB detected protein expression levels of CDK4, CDK6 and cyclin D1, which were key cell cycle proteins for cancer proliferation. Oe-ID2 promoted the expression of CDK4, CDK6 and cyclin D1, while sh-ID2 showed the opposite outcomes (Fig. 3C, D, Fig. S2).

To study the effect of ID2 on thyroid cancer metastasis, transwell migration and wound healing assays were performed. Overexpression of ID2 promoted the migration, while ID2 knockdown inhibited cell migration compared to control (Fig. 3E, F). During cell migration, cells need to adjust their morphology and intercellular interactions to migrate effectively to other locations. EMT plays an important role in the initiation of migration and invasion. At the molecular mechanism level, overexpression of ID2 promoted Sox2, N-cadherin, MMP9 expression and downregulated E-cadherin expression, while knockdown of ID2 showed the opposite results (Fig. 3G, H, Fig. S3). These results indicated that ID2 may be involved in the metastasis of thyroid cancer by acting as a regulatory factor of EMT marker expression. We further examined the effect of ID2 on thyroid cancer EMT and stemness. Cell spheroidization results showed that the volume and number of oe-ID2 clones were the largest (Fig. 3I). This suggested that ID2 can promote the stemness of thyroid cancer cells.

### ID2 promotes thyroid cancer proliferation and EMT in vivo

**Fig. 4:**
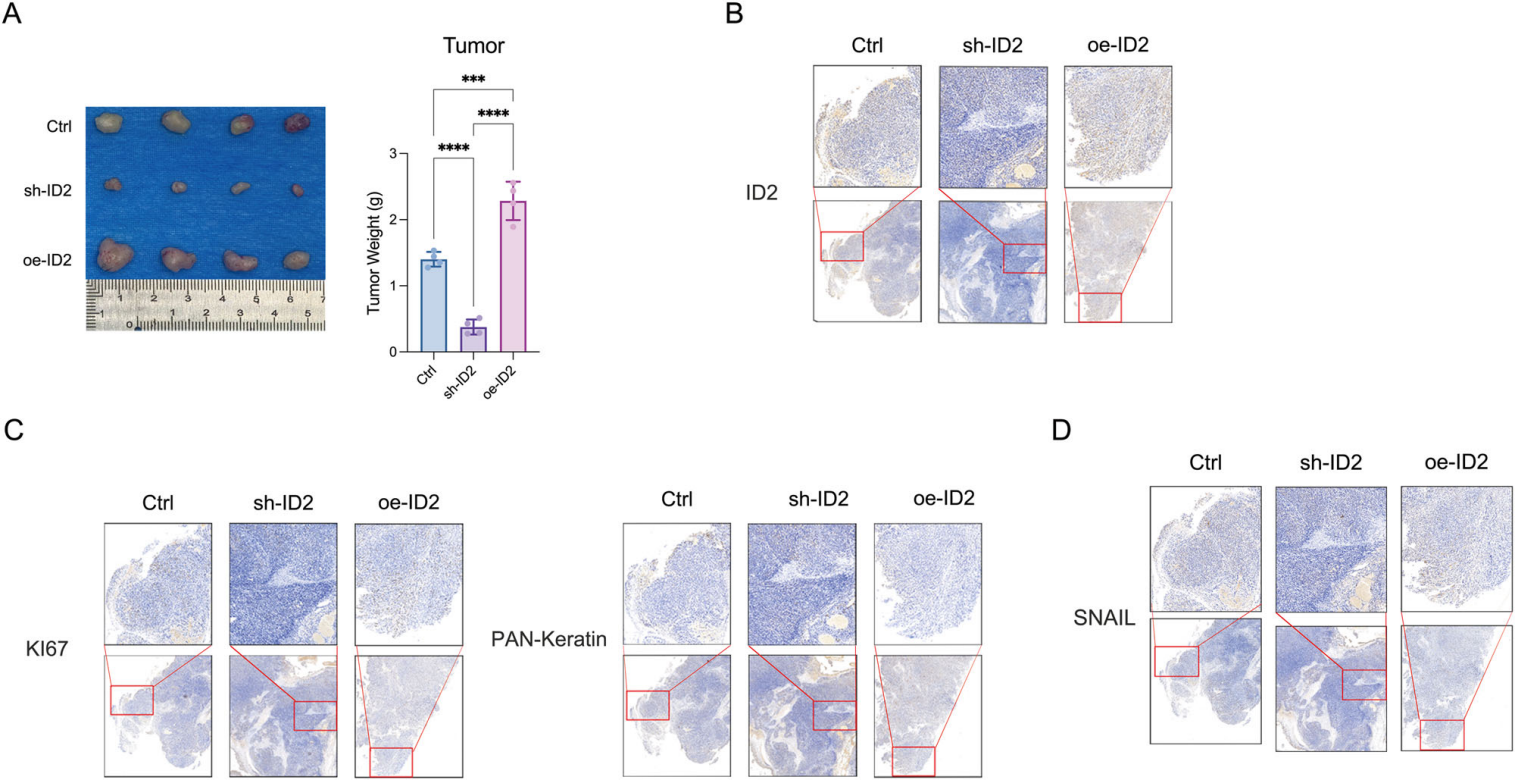
ID2 facilitates cell growth and EMT in vivo. **A** Representative images and weight of xenograft tumors implanted subcutaneously in nude mice. **B** IHC staining confirming the levels of ID2 in tumors transfected with sh-ID2, Ctrl, or oe-ID2. IHC staining demonstrating the levels of growth-related (**C**) and EMT-related (**D**) markers in tumors. *p < 0.05, **p < 0.01, ***p < 0.005, ****p < 0.001

We used oe-ID2, sh-ID2, and control to construct a subcutaneous tumor model of thyroid cancer in nude mice (BALB/c-nu). We observed that oe-ID2 had the fastest proliferation rate in vivo, and the subcutaneous tumor volume and weight were significantly higher than control (Fig. 4A). Similar to the previous results, sh-ID2 had minimal subcutaneous tumors volume and weight (Fig. 4B, C). Hematoxylin - eosin staining (HE) and IHC staining were further performed on subcutaneous tumors, and tumor cells were the most abundant in oe-ID2 (Fig. 4D), with Ki67 and pan-keratins (Fig. 4E) upregulated the most obvious. This further confirmed the promoting effect of oe-ID2 on the proliferation of thyroid cancer in vivo. The expression of stemness marker Snail in subcutaneous tumors was compared by IHC staining (Fig. 4F). The results showed that the stemness marker in oe-ID2 had significantly deeper staining, while the staining in sh-ID2 was the lightest and the range was small. We have demonstrated that ID2 was involved in promoting thyroid cancer stemness both in vivo and in vitro.

### Regulation of the PI3K/Akt signaling pathway by ID2 in thyroid cancer

**Fig. 5:**
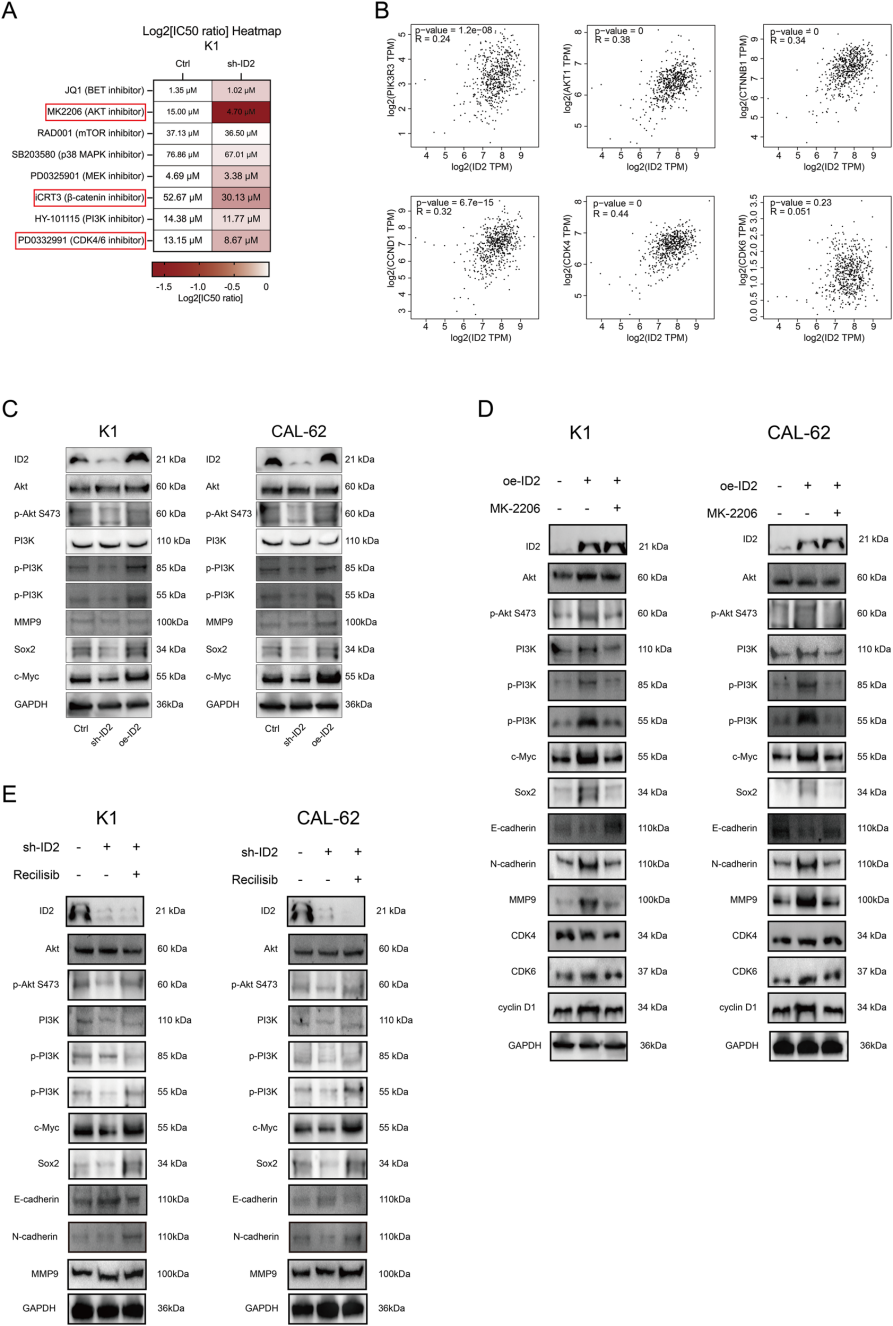
ID2 activates Akt phosphorylation-mediated downstream responses. **A** After transfecting CAL-62 cells with sh-ID2, Ctrl, or oe-ID2, different types of small molecule inhibitors were applied for 48 h. IC_50_ values were analyzed and displayed as a heatmap in comparison to control IC_50_. **B** Correlation analysis in GEPIA between ID2 expression and the expression levels of PI3K, Akt, CTNNB1, CDK4, CDK6, and cyclin D1. **C** WB detectd the expression of PI3K/Akt pathway and downstream target molecules in K1 and CAL-62 transfected with sh-ID2, Ctrl, or oe-ID2. After MK-2206 (AKT inhibitor, **D**) or Recilisib (AKT activator, **E**) treatment, WB detected the expression of PI3K/Akt pathway and downstream molecules

We tested the drug sensitivity (for example IC_50_) of K1 and CAL-62 with NC and sh-ID2 to some small molecule inhibitors of cancer-related pathways (Fig. 5A). The results showed that PI3K/Akt inhibitors further enhanced the viability loss of sh-ID2 cells. Furthermore, the expression relationship between ID2 and PI3K, AKT1, CTNNB1, CDK4, CDK6, and cyclin D1 were analysed by GEPIA (Fig. 5B). The results indicated that ID2 may be involved in regulating intracellular PI3K/Akt signaling pathways.

WB analysis confirmed that the phosphorylation level of Akt was upregulated in oe-ID2, while sh-ID2 inhibited the phosphorylation level of Akt (Fig. 5C, Figs. S4, S7). We hypothesised that ID2 may regulated the activation of PI3K/AKT pathway to promote viability and migration of thyroid cancer. To confirm this hypothesis, NC, sh-ID2 and oe-ID2 cells were treated with Akt inhibitor (MK-2206) and agonist (Recilisib). Oe-ID2increased the expression downstream molecules, and these effects were reversed by MK-2206 treatment (Fig. 5D, Figs. S5, S8, S9). Conversely, the inhibitory effects of sh-ID2 on PI3K/Akt and EMT molecules were also restored due to Recilisib (Fig. 5E, Figs. S6, S10-S11).

In summary, our results demonstrated that ID2 promotes thyroid cancer cell proliferation, migration and EMT by enhancing Akt activation. ID2 may be a promising therapeutic target for thyroid cancer.

## Discussion

The ID protein family includes ID1, ID2, ID3, and ID4, which participate in cellular growth processes and serve as important regulators of cell proliferation and differentiation. The ID family has been shown to be widely involved in the development of various cells and tissues in the body, and is associated with cancer. ID proteins play roles in cell cycle control, cancer development, angiogenesis, and apoptosis in human cancers [32, 33]. ID2 is dysregulated in various cancers. As the role of ID2 varies depending on the cancer type, it is imperative to elucidate how ID2 impacts thyroid cancer progression. In this study, we have identified for the first time that (1) ID2 is highly expressed in thyroid cancer and is associated with shortened overall survival (OS) and disease-specific survival (DSS) in patients. (2) Silencing of ID2 in thyroid cancer cells leads to reduced proliferation, diminished migration ability, dysregulated expression of epithelial-mesenchymal transition (EMT) markers, and suppressed stemness. (3) High co-expression of ID2 and EMT markers in thyroid cancer cells is indicative of poor DSS in patients. (4) ID2 is implicated in the regulation of the PI3K/Akt signaling pathway in thyroid cancer cells.

The ID protein family has been shown to hold significant clinical implications in various cancers. For instance, elevated expression of ID1 is associated with high invasiveness in breast cancer and contributes to the development of hormone-resistant [34]. Both ID1 and ID3 regulate self-renewal of colon cancer stem cells and play a role in resistance to the chemotherapy drug oxaliplatin [7]. Overexpression of ID2 in pancreatic cancer cells promotes cancer cell growth [35], while ID1 is linked to increased tumor angiogenesis in pancreatic cancer [36]. ID2 is abnormally expressed in multiple cancers. It has been reported that ID2 is highly expressed in esophageal squamous cell carcinoma (ESCC) [37], serving as a marker for ESCC metastasis and prognosis. High expression of ID2 has also been observed in colorectal cancer samples, and intraperitoneal injection of ID2 siRNA reduces the growth of colorectal tumors in mice, suggesting that ID2 could be a potential drug target for cancer treatment [38].

It has been reported that ID proteins contribute to the development of tumors by preventing programmed cell death and supporting the survival of tumor cells. Inhibition of pro-apoptosis signals (p21, CDKN2A, and BIM) and up-regulation of anti-apoptosis (BCL-2, BCL-XL) and pro-survival factors (PI3K/Akt, NF-κB) was associated to the aberrant level of ID protein [6, 39, 40]. In this study, we found that oe-ID2 promotes thyroid cancer cell proliferation. Clones of oe-ID2 cells in plates were more in number and larger in size. We discovered that ID2 increased the mRNA and protein expression levels of CDK4, CDK6, and Cyclin D1. These proteins facilitated cell cycle transition and is crucial for the quick growth of cancer. In addition, tthey are frequently identified as the pathway downstream of PI3K-Akt and other signaling pathways that promote malignancy. Other studies have found that cycle-dependent kinase inhibitors CDKN2A and CDKN1A were downregulated as a consequence of ID proteins [6, 41, 42]. It was well-recognized that CDKN2A and CDKN1A had a close association with aging. ID proteins played a dual function in colonization and metastasis. ID proteins were considered to trigger EMT at the primary site to promote invasions, and at the secondary site to increase colonization by switching epithelial state (MET). According to a study, ID proteins act with matrix metalloproteinases (MMPs) and the extracellular matrix to increase invasiveness [43]. Patients with breast cancer who had lymph node metastases typically contained a high expression level of ID1 and low levels of KLF17 [44]. It was also shown that inhibiting ID1 and ID3 did not affect breast cancer cells’ capacity to migrate to the lung [45].

We investigated the role of ID2 in thyroid cancer metastasis. The transwell experiment revealed that oe-ID2 penetrated the membrane more quickly. A higher percentage of the healed region was present in the oe-ID2 wounds. ID2 was able to up-regulate the expression of N-cadherin and down-regulate the expression of E-cadherin, according to PCR and WB results. N-cadherin is a crucial component in the EMT process, and previous research has revealed that ID protein up-regulates it [46–48]. Additionally, ID2 is implicated in the regulation of extracellular moieties, including metalloproteinases. Overexpression of ID2 leads to an upregulation of MMP9, whereas ID2 knockdown demonstrates the contrary effect. These findings suggest that ID2 may modulate MMP9 in thyroid cancer metastasis, as further corroborated by in vivo experiments, thereby providing additional evidence for the facilitation of thyroid cancer proliferation and metastatic potential by ID2. Additionally, we found that in thyroid cancer subcutaneous tumors, ID2 can up-regulate N-cadherin expression while down-regulating E-cadherin. A subgroup of tumor cells that can restart tumor growth is known as cancer stem cells. They fulfill the requirements for tissue stem cell qualities since they are self-renewing and pluripotent. For example, in glioblastoma and colon cancer, the ID protein is involved in the cancer stem cells. In colon cancer stem cells, the combined expression of ID1 and ID3 promotes self-renewal and tumor initiation [7]. ID1, ID2, and ID3 allele deletions in tumors in an in situ model of brain cancer decreased the amount of glioma stem cells, halted tumor growth, and increased the survival time of mice [49]. In vitro, we have observed that ID2 may facilitate cellular proliferation and migration, while also influencing cellular stemness. Western blot results reveal that ID2 induces the expression of proliferation and EMT markers such as CDK4, CDK6, cyclin D1, and N-cadherin. Conversely, knocking down ID2 exerts the opposite effect.

The Akt pathway plays a crucial role in the development and progression of thyroid cancer. The Akt pathway serves as a pivotal intracellular signaling pathway, participating in the regulation of essential biological processes such as cell survival, proliferation, differentiation, and apoptosis. In the context of thyroid cancer, the aberrant activation of the Akt pathway is closely associated with the malignant transformation and heightened invasiveness of cancer cells. Reportedly, the accumulation of PELI1 induced by the loss of miR-30c-5p has been shown to activate the PI3K/Akt pathway, thereby regulating cell proliferation and migration [50]. Additionally, it has been observed that the long non-coding RNA XIST regulates cell proliferation and tumor growth in thyroid cancer through the MET-PI3K-Akt signaling pathway [51]. ID2 regulates tumor progression through distinct molecular signaling pathways. This study has revealed that overexpression of ID2 promotes proliferation and migration of thyroid cancer cells by upregulating the expression levels of p-Akt. Moreover, this effect is partially attenuated by an Akt inhibitor. ID2 holds the potential to serve as a prognostic biomarker and therapeutic target for thyroid cancer patients.

In the majority of human cancers, ID protein expression is dysregulated. ID deregulation in cancer promotes several cancer-specific phenotypes and hence accelerates tumor development. The role of the ID family in the diagnosis and prognosis of thyroid carcinoma is explored in this study. We selected ID2 to be the focus of our additional research, and we investigated how ID2 affected the immune microenvironment, proliferation, metastasis, stemness, and other features of thyroid cancer. Indeed, further understanding of the intricate mechanisms of ID proteins in each tumor type and individual is necessary to pave the way for the development of novel therapeutic targets.

### Supplementary Information

FigureS1

FigureS2

FigureS3

FigureS4

FigureS5

FigureS6

FigureS7

FigureS8

FigureS9

FigureS10

FigureS11

## Data Availability

All data generated or analysed during this study are included in this manuscript. The datasets generated and analysed during the current study are available in the TCGA repository (https://portal.gdc.cancer.gov/), UCSC (https://xenabrowser.net/), TIMER database (https://cistrome.shinyapps.io/timer/), STRING (https://cn.string-db.org/), and GEPIA (http://gepia.cancer-pku.cn/).

## Abbreviation

ID2: Inhibitor of DNA Binding 2
CCK-8: Cell Counting Kit 8
WB: western blot
RT-qPCR: real-time quantitative polymerase chain reaction
IHC: immunohistochemical
PTC: papillary thyroid carcinoma
Akt: protein kinase B
PI3K: phosphatidylinositol 3-kinase
EMT: epithelial-mesenchymal transition
HCC: hepatocellular carcinoma
CRC: colorectal cancer
PBS: Phosphate buffer solution
TCGA: the Cancer Genome Atlas Database
UCSC: University of California Santa Cruz
TIMER: Tumor IMmune Estimation Resource
KM: Kaplan-Meier
ROC: receiver operating characteristic
FDR: false discovery rate
KEGG: Kyoto Encyclopedia of Genes and Genomes
GO: Gene ontology
STRING: search tool for the retrieval of interacting genes/proteins
GEPIA: Gene Expression Profiling Interactive Analysis
OS: overall survival
DSS: disease-specific survival
PPI: protein-protein interaction
