# Supplementary figures and images for: ID2 promotes tumor progression and metastasis in thyroid cancer
Source: Endocrine. 2024 Jan 10;84(3):1051–63. doi: 10.1007/s12020-023-03674-3 (PMC11208273; doi:10.1007/s12020-023-03674-3)

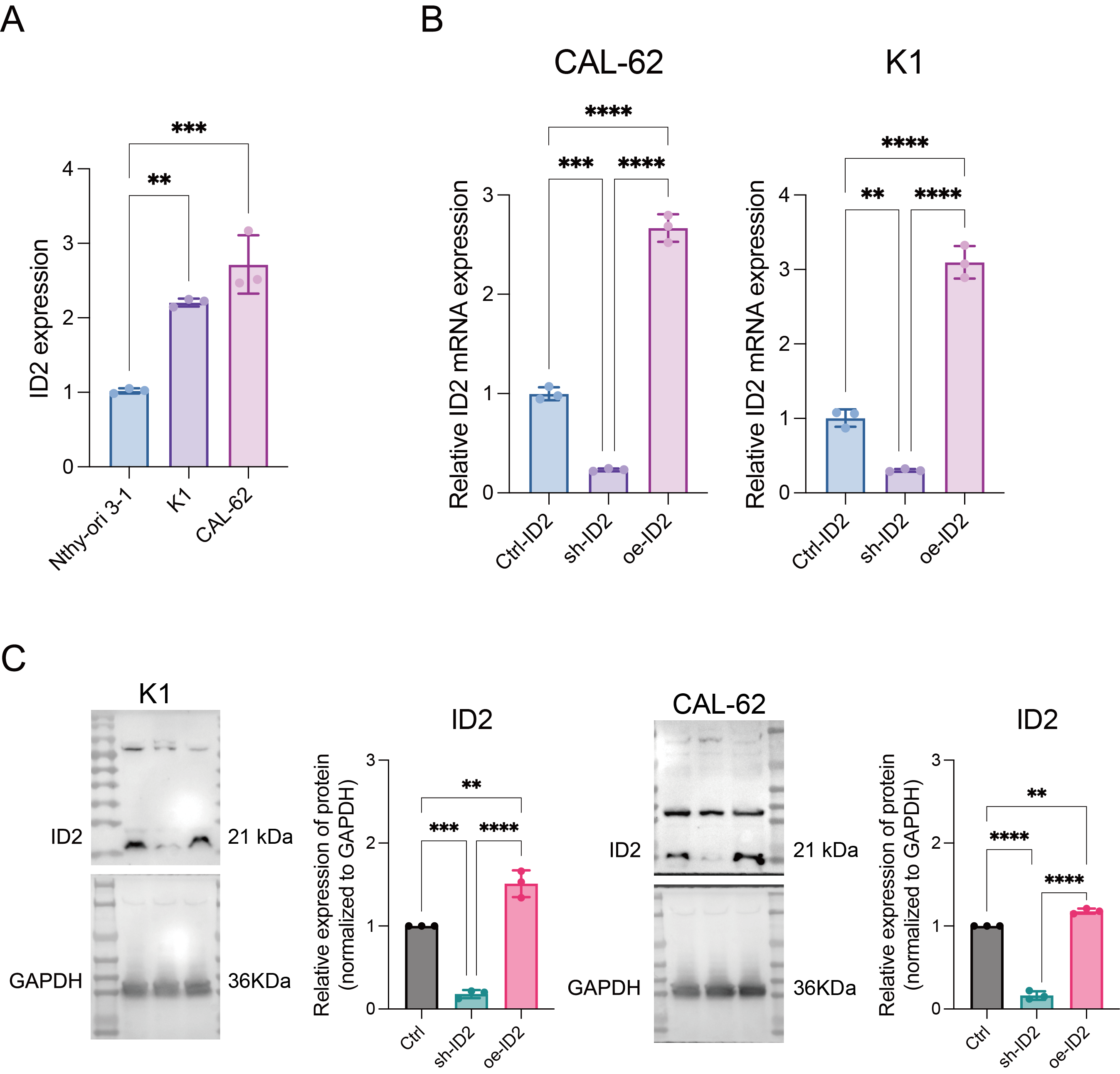

Supplement: Supplementary file 1 — FigureS1 [file 12020_2023_3674_MOESM1_ESM.png]

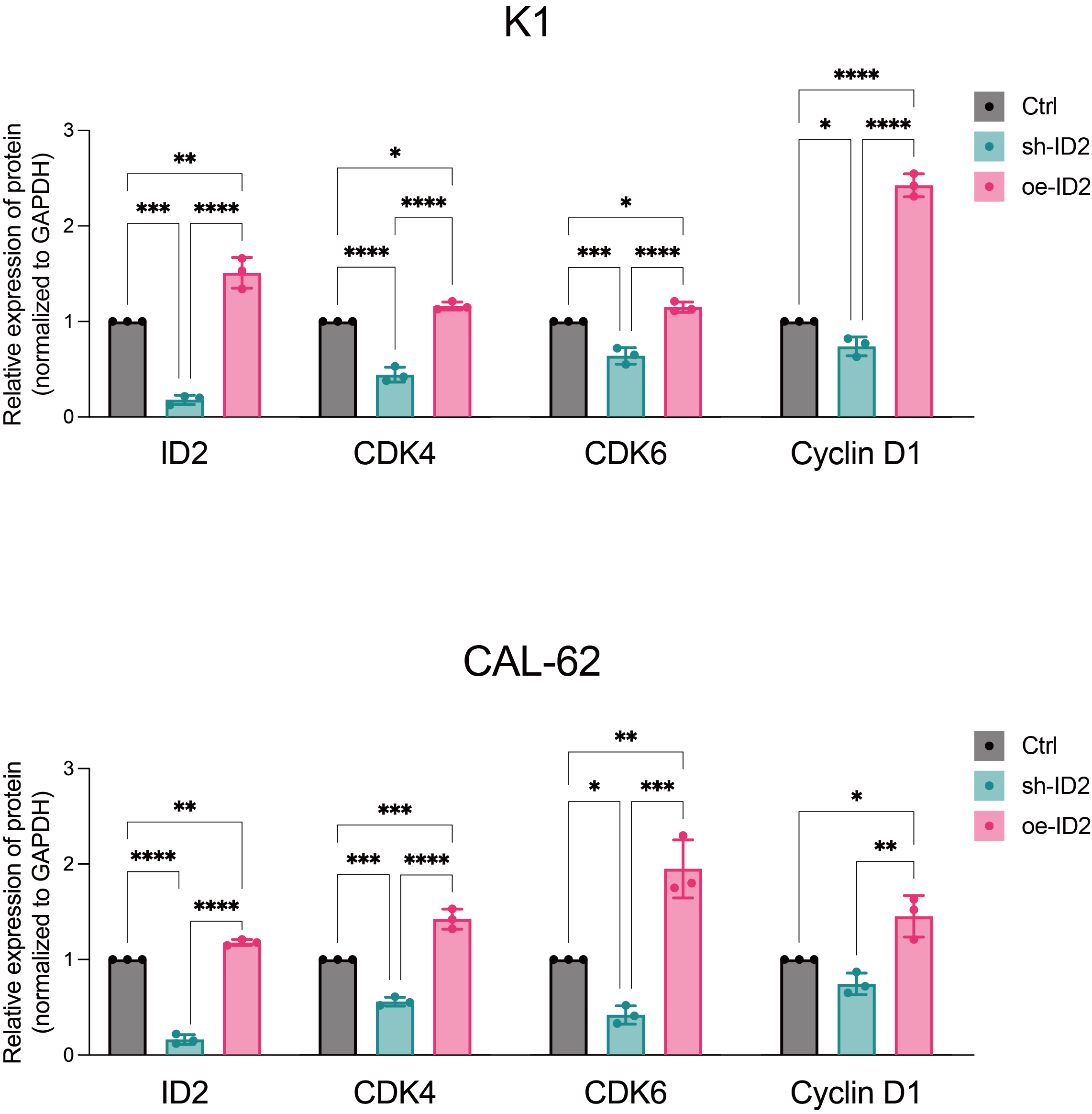

Supplement: Supplementary file 2 — FigureS2 [file 12020_2023_3674_MOESM2_ESM.png]

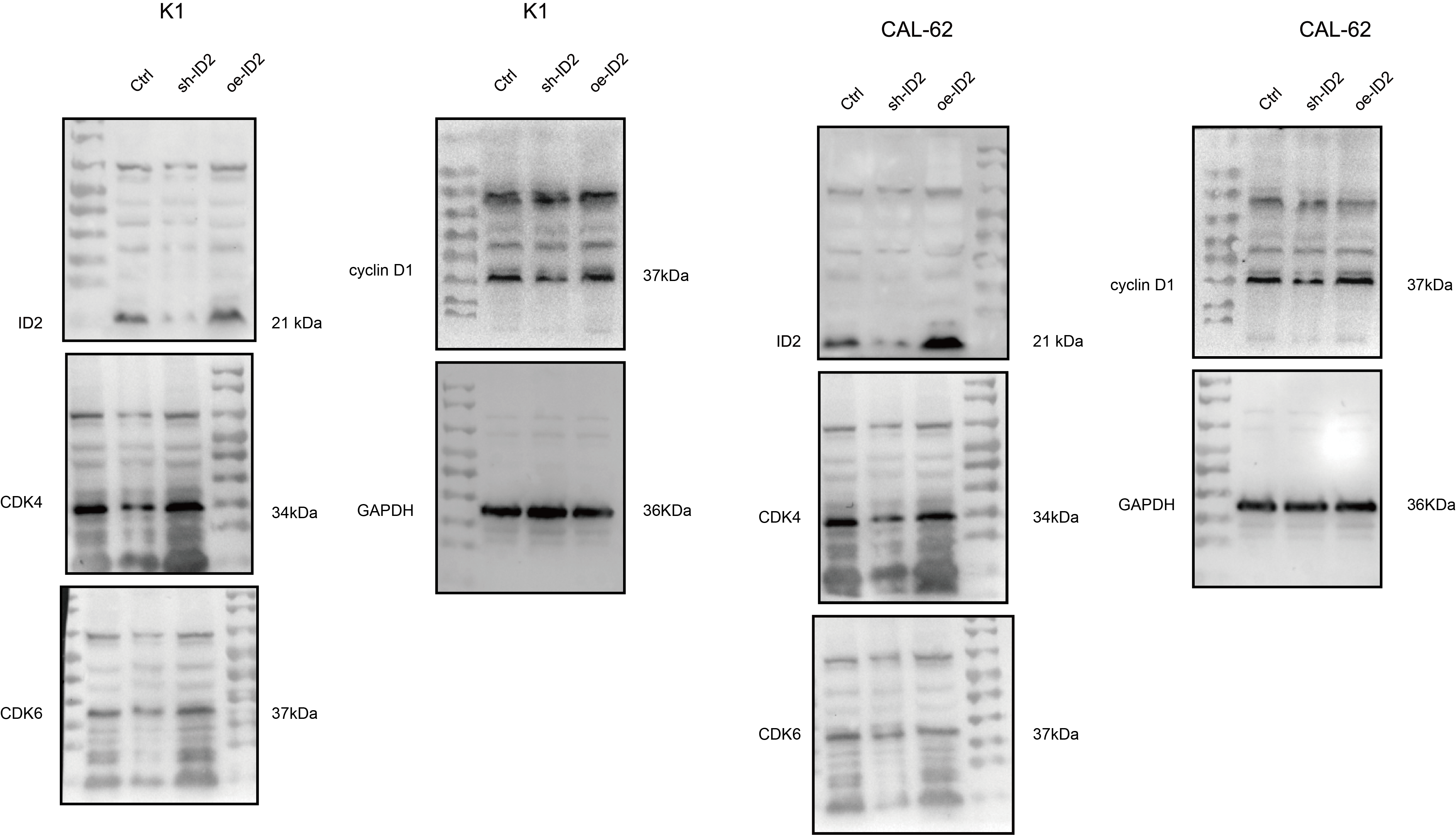

Supplement: Supplementary file 3 — FigureS3 [file 12020_2023_3674_MOESM3_ESM.png]

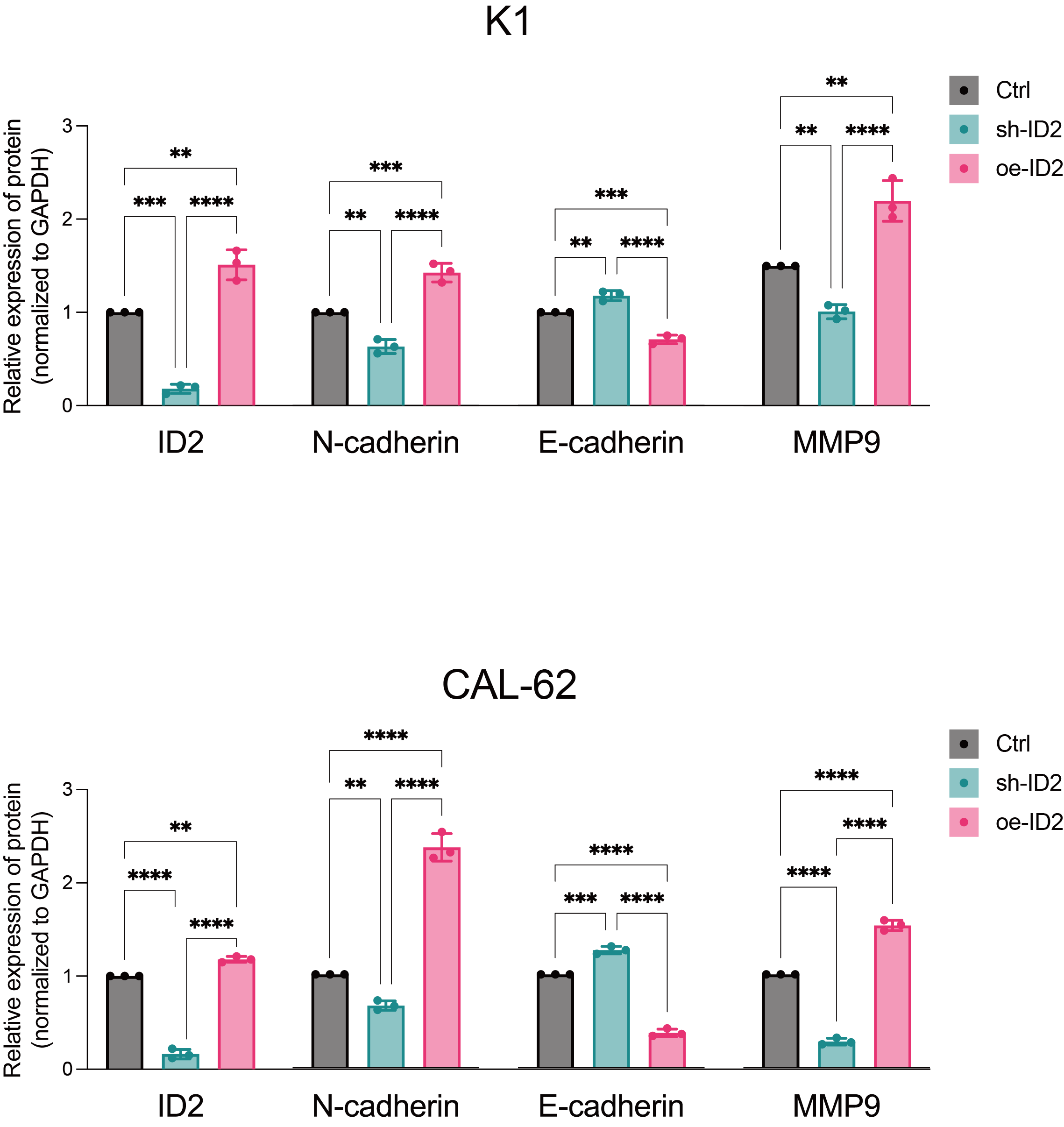

Supplement: Supplementary file 4 — FigureS4 [file 12020_2023_3674_MOESM4_ESM.png]

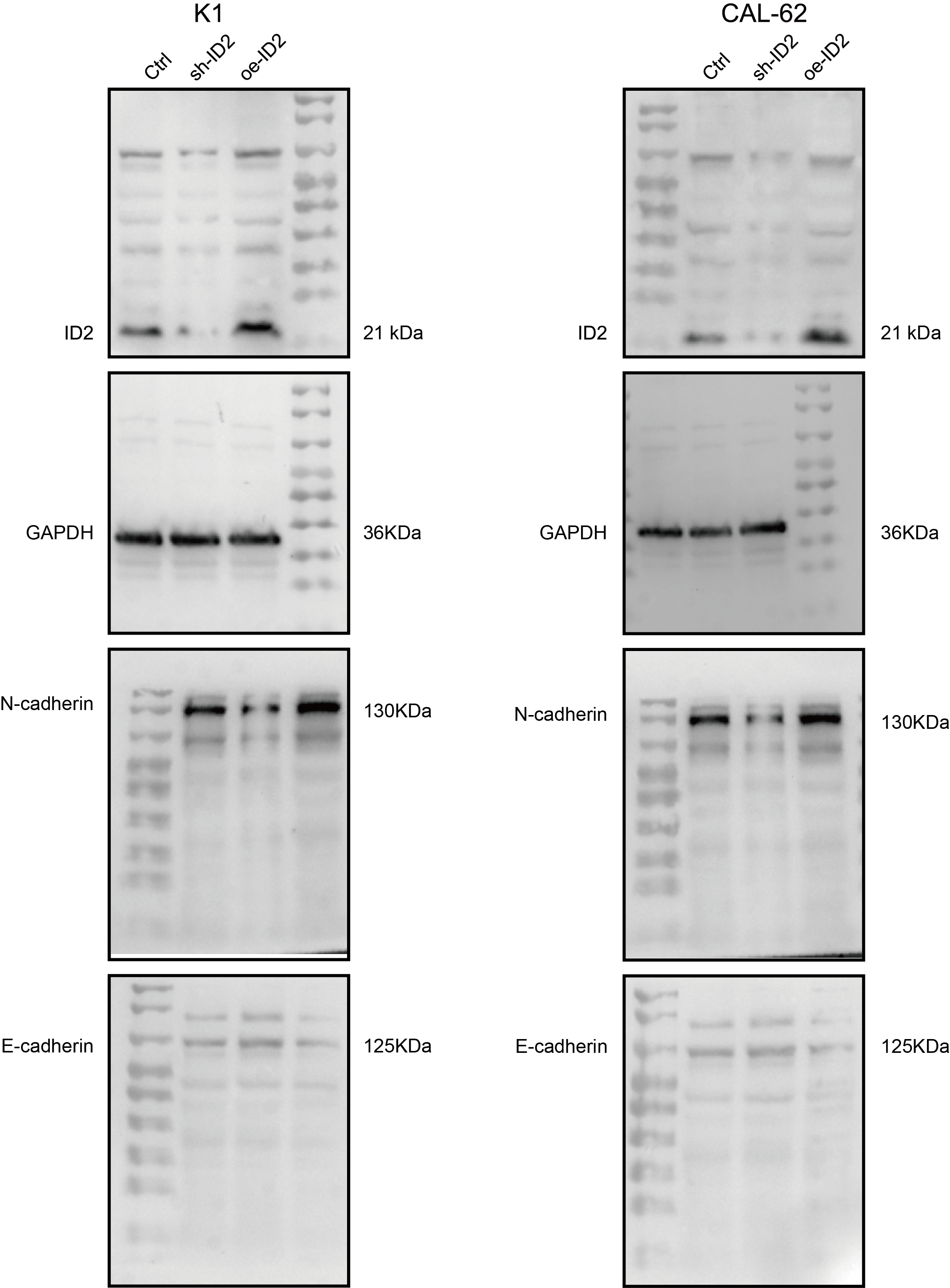

Supplement: Supplementary file 5 — FigureS5 [file 12020_2023_3674_MOESM5_ESM.png]

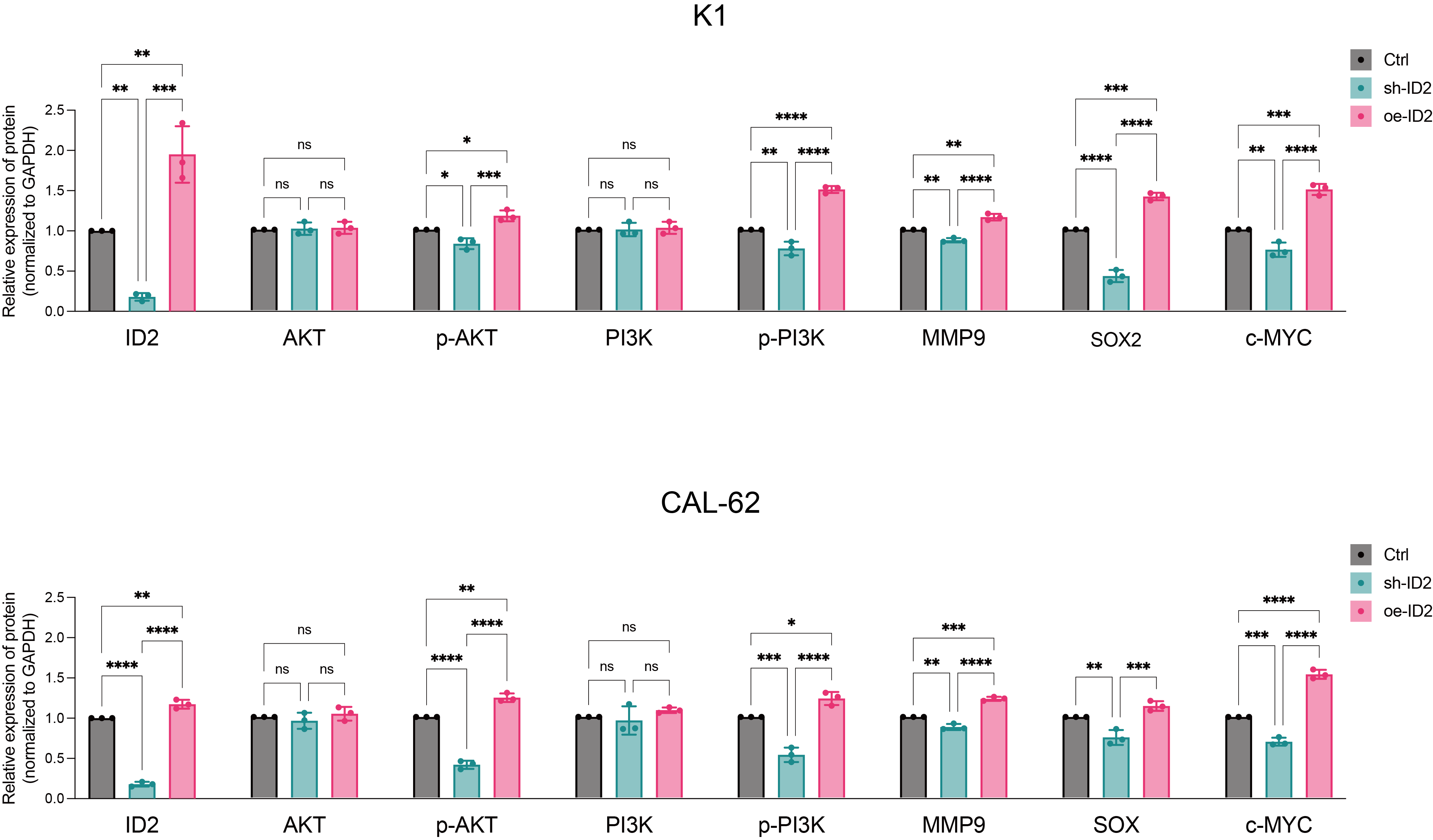

Supplement: Supplementary file 6 — FigureS6 [file 12020_2023_3674_MOESM6_ESM.png]

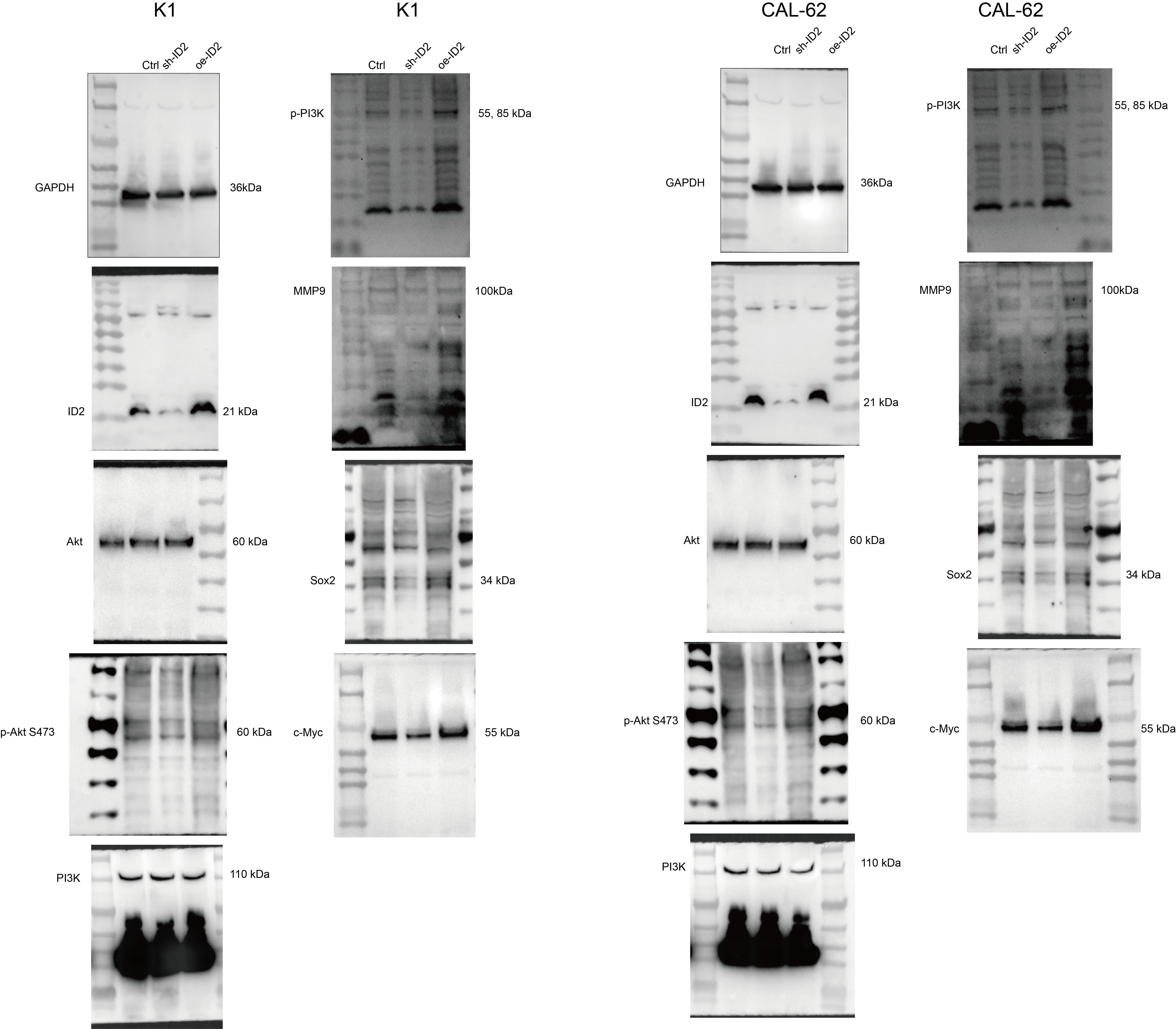

Supplement: Supplementary file 7 — FigureS7 [file 12020_2023_3674_MOESM7_ESM.png]

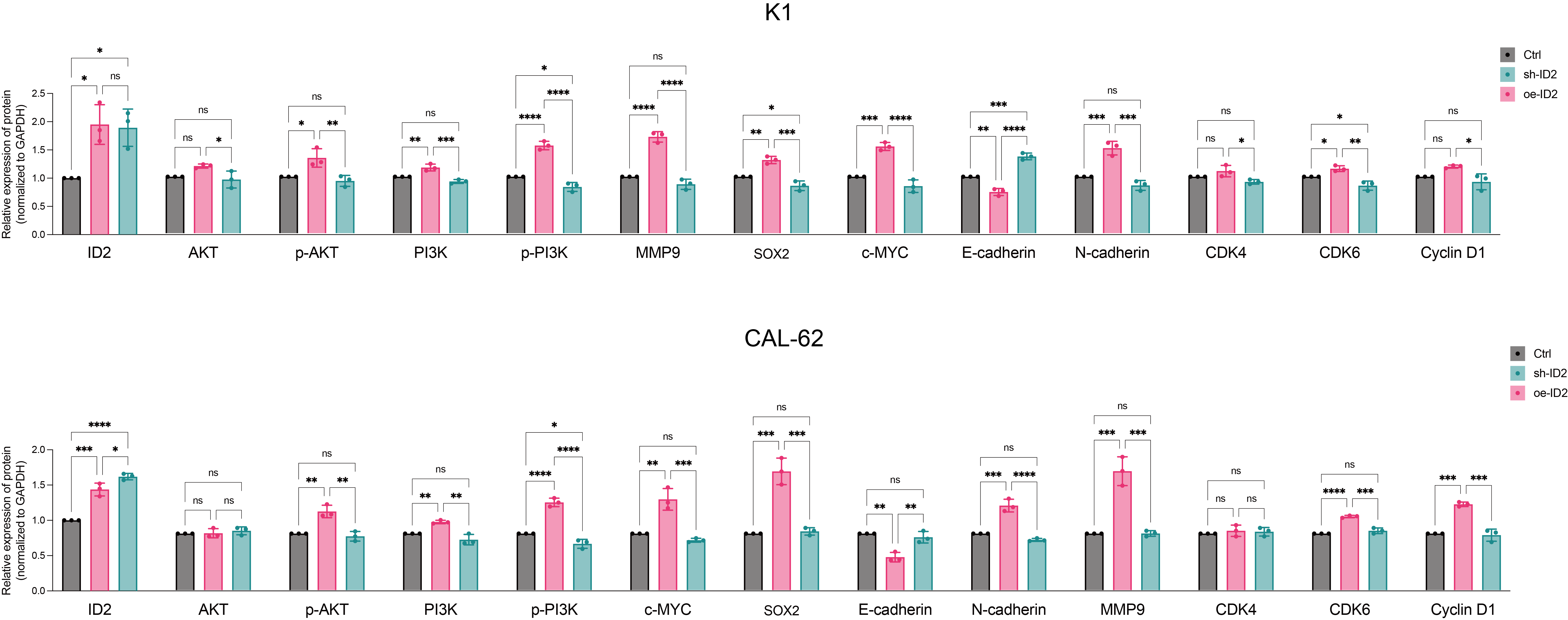

Supplement: Supplementary file 8 — FigureS8 [file 12020_2023_3674_MOESM8_ESM.png]

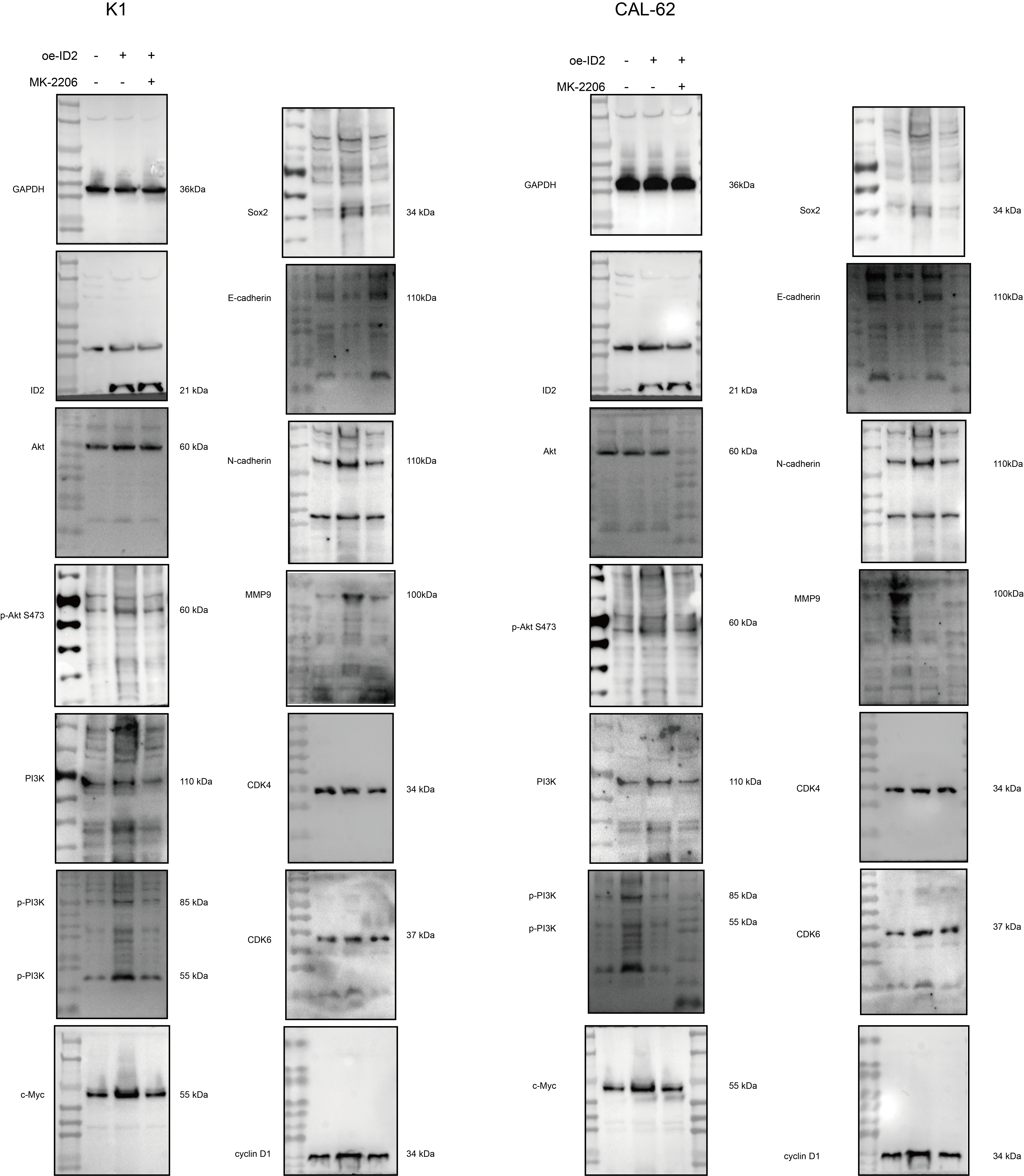

Supplement: Supplementary file 9 — FigureS9 [file 12020_2023_3674_MOESM9_ESM.png]

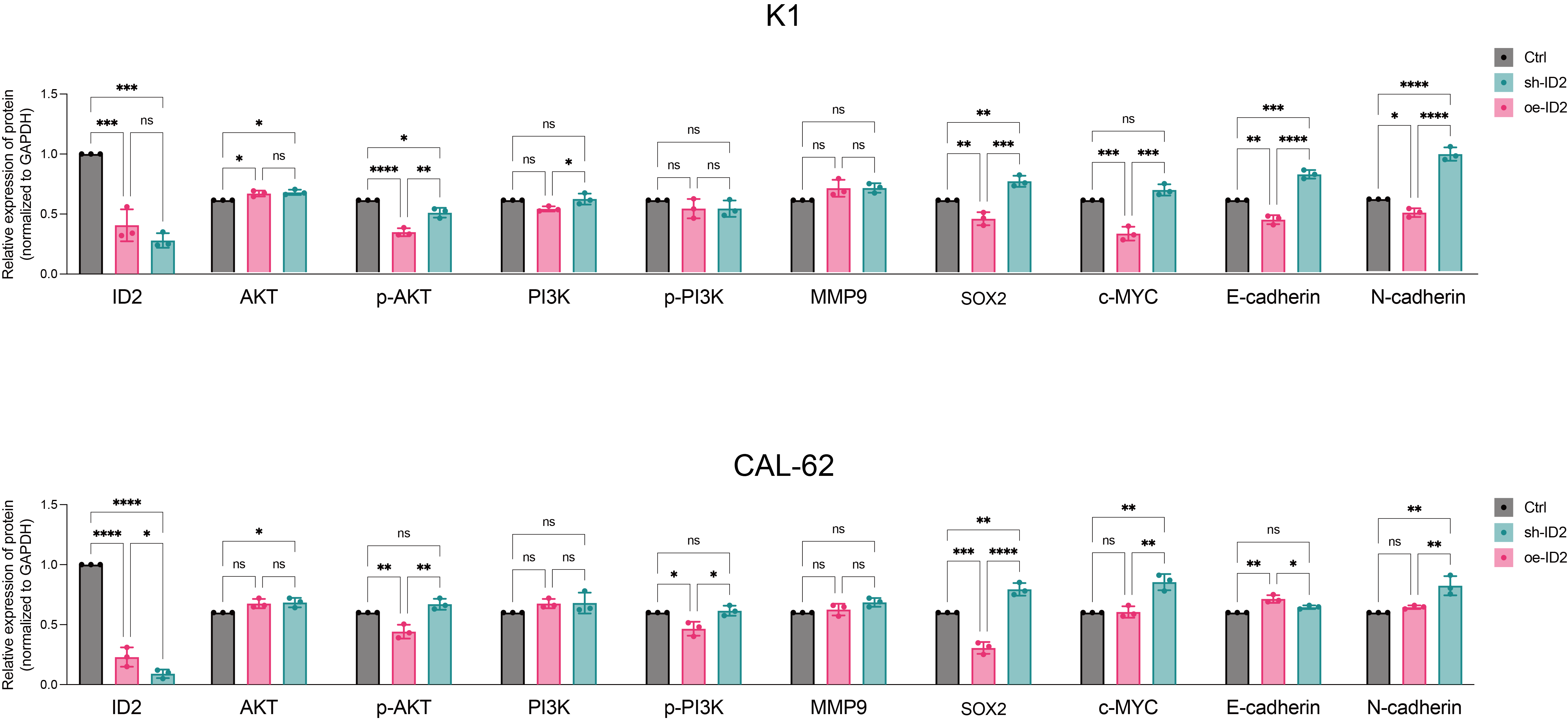

Supplement: Supplementary file 10 — FigureS10 [file 12020_2023_3674_MOESM10_ESM.png]

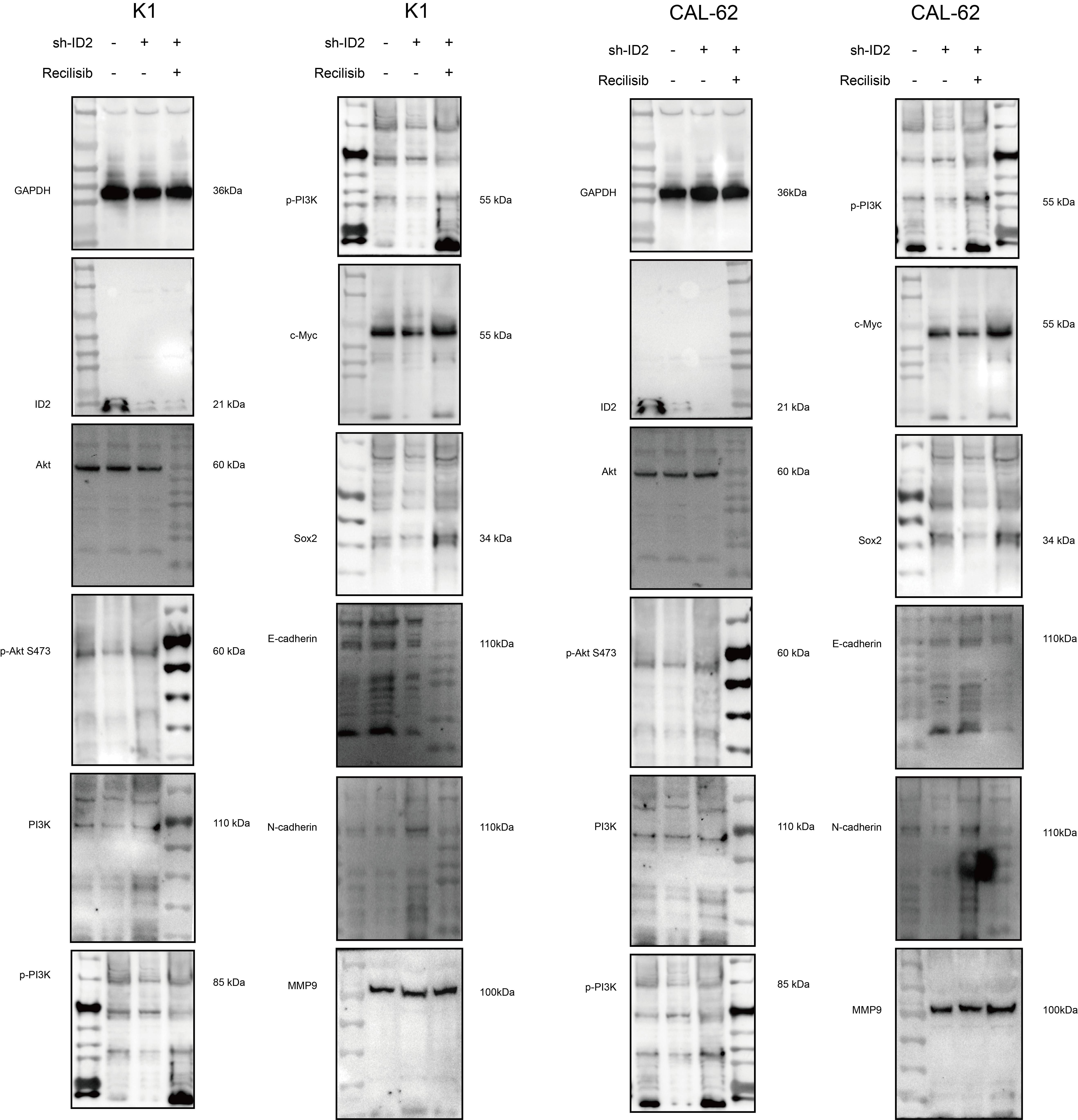

Supplement: Supplementary file 11 — FigureS11 [file 12020_2023_3674_MOESM11_ESM.png]
